# Opioid Therapy in Chronic Pain: Assessment of Clinical Outcomes and Relationships With Endocrine Biomarkers

**DOI:** 10.1002/ejp.70027

**Published:** 2025-04-29

**Authors:** Dalia Abou‐Kassem, Pernille Døssing Kwateng Diasso, Per Sjøgren, Katharina Maria Main, Susanne Dam Nielsen, Geana Paula Kurita

**Affiliations:** ^1^ Multidisciplinary Pain Centre, Department of Neuroanaesthesiology, Pain and Respiratory Support Rigshospitalet Copenhagen University Hospital Copenhagen Denmark; ^2^ Department of Surgery Zealand University Hospital Køge Køge Denmark; ^3^ Section of Palliative Medicine, Department of Oncology Rigshospitalet Copenhagen University Hospital Copenhagen Denmark; ^4^ Department of Growth and Reproduction and EDMaRC Rigshospitalet Copenhagen University Hospital Copenhagen Denmark; ^5^ Department of Clinical Medicine, Faculty of Health and Medical Sciences University of Copenhagen Copenhagen Denmark; ^6^ Department of Infectious Diseases, Rigshospitalet Copenhagen University Hospital Copenhagen Denmark

## Abstract

**Background:**

Long‐term opioid treatment (L‐TOT) may have consequences that are mediated by factors affecting functionality and health‐related quality of life. This study aimed at investigating associations between L‐TOT and clinical outcomes including sustained attention, short‐term and working memory, worst pain intensity, sleep quality, mood, and health‐related quality of life in patients with chronic non‐cancer pain (CNCP). Additionally, the study aimed at exploring whether endocrine biomarkers mediate the relationship between L‐TOT and these outcomes.

**Methods:**

Cross‐sectional study with 82 adult CNCP patients divided into two groups (opioid treated (*n* = 38) and controls not treated with opioids (*n* = 44)). Linear regression analyses assessed associations between L‐TOT, outcome variables, and the mediating effects of endocrine biomarkers.

A Bootstrap approach with 95% confidence intervals was applied to analyse the natural indirect effect.

**Results:**

The opioid group had worse sleep quality (*p* = 0.018), physical functioning (*p* = 0.0186), social functioning (*p* = 0.002), and higher pain intensity (*p* < 0.001) compared with controls. Men in the L‐TOT group experienced worse measures for the same variables, and additionally for anxiety (*p* = 0.028), depression (*p* = 0.040), role physical (*p* = 0.038), role emotional (*p* < 0.001), fatigue (*p* = 0.019), and emotional well‐being (*p* = 0.001). Only the association between L‐TOT and anxiety in men was significantly mediated by total testosterone (β = 1.6, Bias‐Corrected Bootstrap 95% CI: 0.1; 4.1, *p* = 0.045).

**Conclusions:**

CNCP patients in L‐TOT showed significantly poorer outcomes than controls. Only testosterone mediated anxiety in men, indicating a natural indirect effect. Causality and mediating effects of endocrine biomarkers need to be further explored, but the associations are an alert to the potential effects of opioids.

**Significance Statement:**

This study reveals the broader health impacts of opioid use, particularly the role of hormone‐related factors in men, which may be linked to specific adverse effects and consequences. Investigating these hormonal dynamics could lead to improved treatment strategies and outcomes.

## 1 Introduction

Opioids are not recommended as the first choice for treating chronic non‐cancer pain (CNCP) (International Association for the Study of Pain 2018). Notwithstanding, a high prevalence of opioid prescribing for CNCP has been observed over the last few decades, and it is further expected to increase (Andrews et al. 2018; Ekholm et al. 2022; Zimmer et al. 2022). Studies show a high prevalence of opioid prescriptions for CNCP in general populations (1.8%–12.3%) and patients (8.4%–81.2%) across several countries (De Sola et al. 2020). This increase in opioid consumption raises concerns about side effects and long‐term consequences, particularly given the ongoing opioid epidemic in the United States (US) (Center for Disease Control and Prevention 2023).

As opioid treatment for CNCP has increased, knowledge regarding its side effects and consequences has evolved. Beyond the commonly mentioned “classic” side effects as vomiting, constipation, and respiratory depression, the opioid epidemic has highlighted the severe issues of addiction and overdose‐related deaths (Center for Disease Control and Prevention 2023). Additionally, there has been a growing interest in assessing the development of less impressive consequences of L‐TOT, such as cognitive dysfunction, increased pain sensitivity, altered sleep architecture, and mood disturbances (Akhurst et al. 2021; Diasso et al. 2019, 2023; Dublin et al. 2015), all of which may negatively impact health‐related quality of life (HRQoL) (Dillie et al. 2008).

Associations between L‐TOT and poor HRQoL and mood dysfunction are all likely to appear even in connection with successful pain management (Leung et al. 2022; Taylor et al. 2016). However, studies show that opioids can have a positive effect on cognitive function and improve sleep quality as they counteract chronic pain (Cutrufello et al. 2020; Jamison et al. 2003; Rosenthal et al. 2007).

These outcomes may result directly from opioid use or indirectly mediated via the endocrine system, as L‐TOT has been associated with alterations in insulin level, the hypothalamic–pituitary‐gonadal axis (HPG axis), and the hypothalamic–pituitary–adrenal axis (HPA axis) in CNCP patients (Diasso et al. 2021). However, the evidence is weak and primarily related to hypogonadism (Diasso et al. 2021; Gadelha et al. 2022), which can affect sexual function and fertility (Gadelha et al. 2022; Kruljac et al. 2020).

Given the high prevalence of opioid prescriptions for CNCP (De Sola et al. 2020), increasing prevalence of chronic pain (Andrews et al. 2018; Ekholm et al. 2022; Zimmer et al. 2022), and the limited knowledge of long‐term opioid side effects (Diasso et al. 2021), further explorative investigations are necessary. We hypothesized that opioids were associated with poor health‐related outcomes, potentially mediated through endocrine effects. Thus, this study aimed to investigate associations between L‐TOT and sustained attention, short‐term and working memory, worst pain intensity, sleep quality, anxiety, depression, and HRQoL in CNCP patients. Further, we aimed at exploring the mediating effects of endocrine biomarkers on the relationship between L‐TOT and the outcomes.

## 2 Methods

### 2.1 Design, Settings and Ethics

This work is part of a larger cross‐sectional study (Diasso et al. 2023) conducted at the Multidisciplinary Pain Centre (MPC), Rigshospitalet Copenhagen University Hospital, Denmark, from October 2014 to June 2021. The purpose of the larger study was to investigate the effects of L‐TOT on endocrine measures in CNCP patients. This group was compared with a control group, which consisted of CNCP patients who were not in L‐TOT. Eligibility, participation rate, recruitment, and data collection are the same as those carried out in the original study (Diasso et al. 2023). The project was approved by the Danish Data Protection Agency (30–1317) and the Regional Ethics Committee of Copenhagen (H‐1‐2014‐063). This report followed the Guideline for Reporting Mediation Analyses of Randomized Trials and Observational Studies: The AGReMA Statement (Lee et al. 2021).

### 2.2 Sample

The patients were contacted and informed via phone call about the project and had 24 h to reflect on whether they wanted to participate.

Eighty‐two patients were included, and based on a power calculation, we divided them into two groups according to the use of opioids (Diasso et al. 2023). Thirty‐eight patients were treated with opioids with a continuous minimal oral morphine equivalent daily dose (MEDD) of ≥ 30 mg for at least 4 weeks, constituting the opioid group, while 44 patients not using opioids for at least 4 weeks formed the control group.

Patients were between 18 and 65 years old, diagnosed with CNCP (pain for ≥ 6 months), actively treated at the MPC, with at least 6 years of schooling, and were fluent in the Danish language.

In this study, we have defined L‐TOT as stable daily usage for at least 4 weeks.

Patients were excluded if they were treated with glucocorticoids, anti‐hormonal treatment, or any other medication/treatment affecting the endocrine and/or the immune system in the last 6 months, as well as anticonvulsants, antidepressants, and/or anxiolytics consumption for at least 4 weeks. The exclusion of patients taking benzodiazepines and hypnotics is due to the impact of these drugs on the endocrine system. Benzodiazepines may suppress endocrine hormones, e.g., PRL (Grandison 1983), and anticonvulsants may negatively affect the sex hormones (Svalheim et al. 2015). Pregnant or lactating women, patients with active cancer disease, cancer‐related pain, brain disorders (e.g., degenerative nerve diseases, Alzheimer's disease, Parkinson's disease, Epilepsy, dementia and mental retardation) or head trauma within the past 6 months, liver diseases, or renal insufficiency with serum creatinine concentration > 140 μmol/L were also excluded. Head trauma was excluded due to its possible impact on cognitive function, and therefore to avoid interference with the results of the cognitive tests. The frequent use of paracetamol and/or nonsteroidal anti‐inflammatory drugs (NSAIDs) among CNCP patients was deemed to have minimal influence on the endocrine system and was not considered an exclusion criterion. Our criteria were designed to exclude participants with conditions or factors known to influence the outcomes independently of the intervention.

### 2.3 Assessments

All patients met in the MPC from 09.00 AM to 12.00 PM. The inclusion took place in a room where the patient had to fill in informed consent and questionnaires about their sociodemographics, pain characteristics, opioid treatment, other medications, smoking habits, alcohol consumption, height, and weight. Information about the type of pain was obtained from the patient record. In addition, the patient also had to fill in questionnaires about their pain, sleep quality, mood as well as perform cognitive tests. Blood samples were collected between 9:45 AM and 12:45 PM, analyzed, and stored in a research biobank at Rigshospitalet.

Assessments included characterization variables (sociodemographic), an independent variable, dependent variables, and mediator variables. The independent variable was opioid treatment (Y/N). The dependent variables were sustained attention, short‐term memory/working memory, worst pain intensity, sleep quality, mood (anxiety and depression), and health‐related quality of life (HRQoL), assessed through the following tests and questionnaires: Continuous Reaction Time (CRT), Digit Span Test (DST), Numeric Rating Scale (NRS), The Pittsburgh Sleep Quality Index (PSQI), Hospital Anxiety and Depression Scale (HADS), and RAND 36‐Item Health Survey (RAND SF‐36). The mediators were endocrine measurements based on previously published findings (Diasso et al. 2023) and included prolactin (PRL), sex hormone‐binding globulin (SHBG), total testosterone (TT), free testosterone (fT), dehydroepiandrosterone sulphate (DHEAS), insulin‐like growth factor 1 (IGF‐1), and IGF‐1 standard deviation score (IGF‐1 SDS).

#### 2.3.1 Assessments of Cognitive Function

Two neuropsychological instruments assessed sustained attention and short‐term/working memory.

The Continuous Reaction Time (CRT) computer test measured sustained attention (Echo program, version 1.3, Bitmatic, Denmark). Patients were instructed to press a button upon hearing 100 auditory signals (500 Hz, 90 dB) presented at varying intervals, with the reaction time recorded in milliseconds (Lauridsen et al. 2013). The program scores the result according to three percentiles (10th, 50th, and 90th), with faster values indicating better performance. Cutoff scores proposed by the program developers for an adequate CRT are: 10th percentile < 165 milliseconds, 50th percentile < 195 milliseconds, and the 90th percentile < 250 milliseconds. Faster values indicate better performance (Lauridsen et al. 2013).

The Digit Span Test (DST) measured short‐term memory and working memory. Patients repeated a series of numbers of increasing lengths (3–9 digits) in forward and backward order. Scores range from 0 to 14 for each phase, with higher scores indicating better performance. Cutoff score for good performance: forward order = 7 (SD = 2), and for backward = 4 (SD = 1). Higher scores indicate better performance (Black 1986; Iverson and Tulsky 2003).

#### 2.3.2 Pain Intensity

The numerical rating scale (NRS) assessed the intensity of the worst pain experienced in the past 24 h, ranging from 0 (no pain) to 10 (highest level of pain) (Haefeli and Elfering 2006).

#### 2.3.3 Sleep Quality

The Pittsburgh Sleep Quality Index (PSQI) evaluated sleep quality and disturbances over 1 month, with seven components (subjective sleep quality, sleep latency, sleep duration, habitual sleep efficiency, sleep disturbances, use of sleep medication, daytime dysfunction) summing to a global sleep score from 0 to 21. Lower scores indicated better sleep quality (Buysse et al. 1989).

#### 2.3.4 Anxiety and Depression

The Hospital Anxiety and Depression Scale (HADS) assessed mood through seven questions for anxiety and another seven questions for depression. A score above 7 indicated potential anxiety or depression, respectively (Bjelland et al. 2002; Zigmond and Snaith 1983).

#### 2.3.5 Health‐Related Quality of Life

RAND 36‐Item Health Survey 1.0 (RAND SF‐36) assessed HRQoL, including functional ability, with 36 items organised into eight components (bodily pain, general health, mental health, physical functioning, role limitations due to emotional problems, role limitations due to physical health, social functioning, and vitality). Scores for domains ranged from 0 to 100, with higher scores indicating better HRQoL (Hays et al. 1993).

#### 2.3.6 Endocrine Biomarkers

The selected endocrine biomarkers have previously been reported by Diasso et al. (2023). Diasso et al. (2023) reported high PRL and low IGF‐1 SDS among all patients in L‐TOT, while TT, fT (calculated from the concentration of TT), SHBG, DHEAS, and IGF‐1 demonstrated group differences only in men (low TT, low fT, high SHBG, low DHEAS and low IGF‐1). The analysis of other endocrine biomarkers did not show differences and they were not included (Diasso et al. 2023). All the endocrine biomarkers were treated as continuous data (Diasso et al. 2023). All blood samples were analysed at the Department of Clinical Biochemistry, Rigshospitalet, except for IGF‐1 which was analysed at the Department of Growth and Reproduction, Rigshospitalet. Plasma concentration of TT, androstenedione, 17‐hydroxyprogesterone, and DHEAS were measured by LC‐MS/MS (Roche), SHBG by Immulite 2000 (Roche) and IGF‐1 by chemiluminescence assay (IDS‐iSYS) in the Multidiscipline Automated Analyser (IDS‐iSYS, Poeilly‐en Anxoic). PRL was analyzed by B•R•A•H•M•S′ Kryptor instrument.

### 2.4 Statistical Analysis

All analyses were performed in SAS v.9.4 (SAS Institute Inc). A power calculation has been conducted for CRT, based on available data related to opioid use and cognitive performance (Sjøgren et al. 2005), using a Welch–Satterthwaite t‐test. A mean difference of 6.8 ms in reaction time (CRT) between the groups (CRT_50th was 29.3 ms with a SD = 12.2, while the control group had a mean = 22.5 ms and a SD = 5.0) required a sample size of 32 patients per group to detect a significant difference, with a two‐sided 5% significance level and 80% power. A power calculation based on any endocrine outcome was not performed due to the lack of adequate published data.

Few patients failed to complete some of the measures (control group: CRT and DST backward = 1 patient, PSQI =3 patients, HADS = 2 patients, RAND SF‐36 = 1 patients; opioid group: PSQI = 2 patients, RAND SF‐36 physical functioning = 1 patients). Endocrine measures were also missing for PRL = 1 patient and IGF‐1 (SDS) = 8 patients. The analyses were performed with the retained complete data.

Sociodemographic characteristics that could interfere with endocrine analyses, along with differences between groups regarding health behaviors (Diasso et al. 2023), were accounted for, and the analyses were adjusted for sex, age, body mass index (BMI), smoking habit, and alcohol consumption. Sensitivity analysis of sub‐groups regarding pain intensity and opioid dose (below and above 90 mg morphine equivalent daily) did not reveal differences and were not considered for analysis adjustment. The rationale for using sensitivity analyses was according to the cut‐off between low and high opioid doses (Dowell et al. 2016; Food and Drug Administration US 2021).

Descriptive data of the groups' outcomes were analysed and compared using linear regressions to examine associations between outcomes (dependent variables) and opioid use (Y/N). Two sets of analyses were conducted: one for all patients and one only for men in both groups, in accordance with Diasso et al. (2023) findings. Significance levels were classified as weak evidence for associations between L‐TOT and the variables (0.01 < *p* < 0.05), moderate evidence (0.001 < *p* ≤ 0.01), and strong evidence (*p* ≤ 0.001) (Ganesh and Cave 2018). Additionally, we analysed the data using the Minimal Clinically Important Difference (MCID) thresholds to interpret the clinical significance of statistically significant associations. Considering the variability in MCIDs across instruments, populations, and the limited literature available for mixed populations of patients with CNCP, we adapted and adopted the following thresholds: ≥ 5.0 points for the subscales of the RAND SF‐36, > 2.1 points for HADS—anxiety, and > 2.5 points for HADS—depression (Grönkvist et al. 2024). For sleep quality, assessed using PSQI, MCID was ≥ 3 points (Buysse et al. 2011).

**FIGURE 1:**
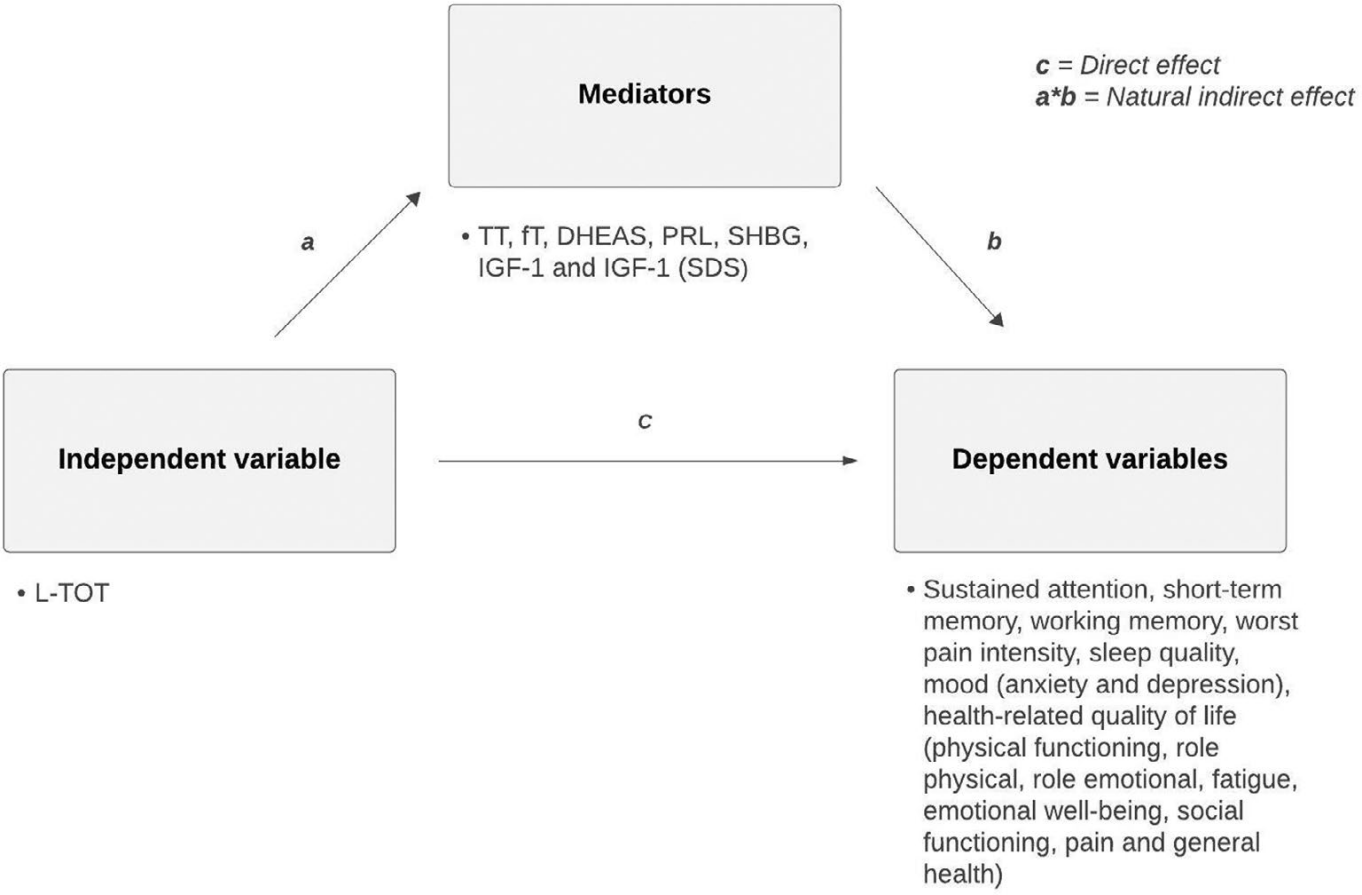
Mediation model.

The mediation analysis followed the Baron & Kenny' steps (Baron and Kenny 1986). Significant associations between opioid exposure (L‐TOT) and outcome variables from the previous analyses (significant dependent variables) were repeated, controlling for endocrine parameters (mediator, Figure 1). Two sets of analyses were performed: one for all patients with the potential mediators PRL and IGF‐1 SDS, and one only for men with TT, fT, SHBG, DHEAS, and IGF‐1. A mediation effect was indicated if the relation between L‐TOT and patient outcomes was not significant when controlling for the mediator (*p* > 0.05), suggesting that the effect of opioids might be mediated by the endocrine biomarker. Partial mediation results were not analyzed due to the study's exploratory nature.

**FIGURE 2:**
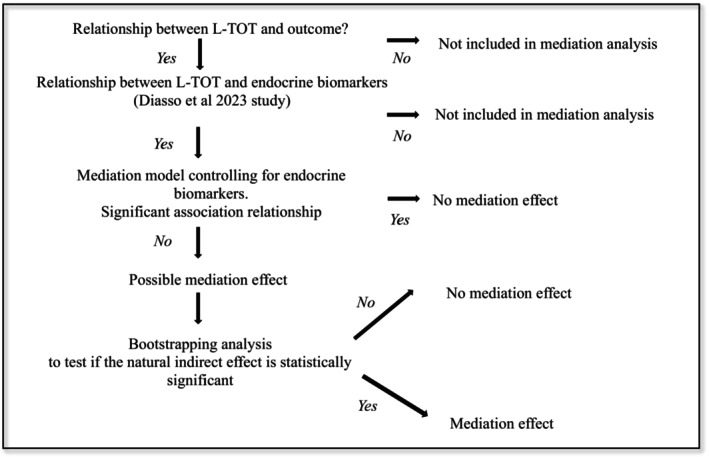
Mediation analysis process.

A regression‐based approach (SAS macro) estimated the natural indirect effects of opioid exposure (L‐TOT) on outcomes (dependent variables) through the mediator (Lee et al. 2021). A 95% bias‐corrected confidence interval (95% CI) was calculated using bootstrapping with 5000 resamples (Mallinckrodt et al. 2006) for significant variables in the previous step. In addition, percentile bootstrap confidence intervals were checked. Confidence intervals that did not contain 0 and had *p* < 0.05 indicated significant mediators. The mediation analysis process can be observed on Figure 2.

## 3 Results

### 3.1 Sample Characteristics

There were significant statistical differences between the two groups regarding smoking, alcohol intake, and BMI. Patients in the opioid group were more likely to smoke, less likely to consume alcohol, and had a higher BMI (Table 1). The opioid and control groups did not show significant statistical differences in terms of sex, age, and pain diagnoses.

**TABLE 1:** Characteristics of the study population.

Variables	Opioid group, *n* = 38	Control group, *n* = 44	*p*
Sex, *n* (%)			
Male	22 (57.9)	18 (40.9)	0.125
Female	16 (42.1)	26 (59.1)	
Age (years)			
Mean (SD)	48.5 (±10.7)	43.3 (±12.3)	0.059
Smoking, *n* (%)			
Yes	22 (57.9)	14 (31.8)	0.018
No	16 (42.1)	30 (68.2)	
Alcohol, *n* (%)			
Yes	14 (36.8)	26 (59.1)	0.044
No	24 (63.16)	18 (40.91)	
BMI			
Mean (SD)	26.6 (±4.3)	24.6 (±4.2)	0.02
Pain characteristics, *n* (%)			
Neuropathic	9 (23.7)	13 (29.6)	0.681
Nociceptive somatic	10 (26.3)	12 (27.3)	
Nociceptive visceral	0 (0)	0 (0)	
Nociceptive visceral and neuropathic	1 (2.63)	0 (0)	
Nociceptive somatic and neuropathic	18 (47.4)	19 (43.2)	

*Note:* Extracted from Diasso et al. (2023).

In the opioid group, most patients were being treated with morphine (*n* = 14, 36.8%) while the rest were being treated with tramadol (*n* = 10, 26.3%), methadone (*n* = 9, 23.7%), oxycodone (*n* = 8, 21.1%), fentanyl (*n* = 2, 5.3%), tapentadol (*n* = 1, 2.6%) and buprenorphine (*n* = 1, 2.6%). The treatment with opioids was a mix of immediate‐ and sustained‐release formulations. Mean MEDD was estimated to be 136.9 mg (±122.1) with a median of 95 mg (SEM = 19.81).

### 3.2 Descriptive Analysis of the Groups' Outcomes

In the opioid group, some dependent variables showed poorer mean scores compared to normal data cut‐offs. The patients had slower reaction time on the CRT (50th percentile = 212.5 ± 132.6 ms, 90th percentile = 290.6 ± 221.0 ms), fewer correct answers on the DST forward (5.5 ± 1.7), and a score indicating possible anxiety (8.2 ± 4.4). In addition, there was high worst pain intensity (6.8 ± 2.1), poor sleep quality (12.6 ± 4.5), and 7 out of 8 HRQoL indicators with mean scores between 12.5 and 42.2 (lowest scores for role physical = 12.5 ± 24.5 and pain = 18.8 ± 19.0) (Table 2).

**TABLE 2:** Descriptive analysis of the groups' outcomes.

Group	Variable	*N*	Mean	SD	Median	Minimum	Maximum
Control (*n* = 44)							
Sustained attention (CRT)	10th percentile	43	145.1	22.1	145.0	119.0	257.0
	50th percentile	43	184.8	53.1	175.0	137.0	434.0
	90th percentile	43	268.8	183.1	232.0	158.0	1313.0
Short‐term/working memory (DST)	Forward	44	5.9	1.5	6.0	3.0	10.0
	Backward	43	5.1	1.5	5.0	2.0	8.0
Pain intensity	Worst	44	6.2	2.2	7.0	0	10.0
Sleep (PSQI)	Global score	41	9.6	4.3	10.0	1.0	17.0
Mood (HADS)	Anxiety	42	8.2	4.8	7.5	0	19.0
	Depression	42	5.3	4.2	5.0	0	13.0
Health‐related Quality of Life (RAND SF‐36)	Physical functioning	43	58.7	25.0	60.0	5.0	100.0
	Role physical	43	28.5	37.6	0	0	100.0
	Role emotional	43	62.0	42.2	66.7	0	100.0
	Fatigue	43	38.1	24.5	30.0	5.0	100.0
	Emotional well being	43	65.0	21.3	68.0	12.0	100.0
	Social functioning	43	61.3	26.8	62.5	0	100.0
	Pain	43	37.9	21.9	32.5	0	90.0
	General health	43	48.4	27.7	45.0	10.0	100.0
Opioid (*n* = 38)							
Sustained attention (CRT)	10th percentile	38	165.0	78.4	140.5	112.0	535.0
	50th percentile	38	212.5	132.6	172.5	133.0	897.0
	90th percentile	38	290.6	221.0	240.5	163.0	1508.0
Short‐term/working memory (DST)	Forward	38	5.5	1.7	5.0	3.0	10.0
	Backward	38	4.8	1.3	4.0	3.0	7.0
Pain intensity	Worst	38	6.8	2.1	7.0	2.0	10.0
Sleep (PSQI)	Global score	36	12.6	4.5	14.0	2.0	18.0
Mood (HADS)	Anxiety	38	8.2	4.4	8.5	0	19.0
	Depression	38	6.3	3.8	6.0	0	13.0
Health‐related Quality of Life (RAND SF‐36)	Physical functioning	37	38.5	22.1	40.0	0	90.0
	Role physical	38	12.5	24.5	0	0	100.0
	Role emotional	38	42.2	38.5	33.3	0	100.0
	Fatigue	38	28.2	20.7	22.5	5.0	90.0
	Emotional well being	38	58.2	19.5	56.0	24.0	96.0
	Social functioning	38	37.8	28.0	25.0	0	100.0
	Pain	38	18.9	19.0	17.5	0	77.5
	General health	38	39.1	25.3	35.0	5.0	90.0

Abbreviations: CRT: continuous reaction time; DST: digit span test; HADS: Hospital Anxiety and Depression Scale; PSQI: Pittsburgh Sleep Quality Index; RAND SF‐36: 36‐Item Short Form Survey.

The control group also had a score indicating anxiety (8.2 ± 4.8). In addition, they also showed high worst pain intensity (6.2 ± 2.2), poor sleep quality (9.6 ± 4.3), and 4 out of 8 HRQoL indicators with mean scores between 28.5 and 48.5 (lowest score to role physical = 28.5 ± 37.6, pain = 37.9 ± 21.9, and fatigue = 38.1 ± 24.5) (Table 2).

### 3.3 Outcomes Comparisons Between Opioid and Control Groups (Including all Patients)

Using linear regression analysis to investigate the six sets of outcome variables between the two groups, two were found to be significant: sleep and HRQoL. Patients in L‐TOT had higher scores in the global sleep measure of PSQI (*p* = 0.018, weak evidence); however, this did not reach the MCID threshold. They also had lower scores in physical functioning (*p* = 0.0186, weak evidence), social functioning (*p* = 0.002, moderate evidence), and pain (*p* < 0.001, strong evidence) on the RAND SF‐36 subscales (Table 3). These differences surpassed the MCID thresholds, demonstrating clinical significance.

**TABLE 3:** Adjusted linear regression analysis to compare outcomes between groups—all patients.

Outcomes	Group	*n*	Estimate	95% CI		*p*
Sustained attention (CRT)						
10th percentile	Opioid	38	14.9	−13.3	43.1	0.295
	Control	43				
50th percentile	Opioid	38	17.3	−32.1	66.7	0.487
	Control	43				
90th percentile	Opioid	38	−6.4	−106.3	93.6	0.900
	Control	43				
Memory (DST)						
Forward	Opioid	38	−0.2	−0.98	0.57	0.600
	Control	44				
Backward	Opioid	38	−0.2	−0.9	0.5	0.486
	Control	43				
Worst pain intensity	Opioid	38	0.7	−0.4	1.8	0.199
	Control	44				
Sleep (PSQI)						
Global score	Opioid	36	2.8	0.5	5.1	**0.018**
	Control	41				
Mood (HADS)						
Anxiety	Opioid	38	0.0	−2.3	2.4	0.997
	Control	42				
Depression	Opioid	38	1.0	−1.1	3.1	0.335
	Control	42				
Health‐related Quality of Life (RAND SF‐36)						
Physical functioning	Opioid	37	−14.0	−25.7	−2.4	**0.0186**
	Control	43				
Role physical	Opioid	38	−15.3	−31.7	1.2	0.068
	Control	43				
Role emotional	Opioid	38	−13.3	−33.1	6.5	0.185
	Control	43				
Fatigue	Opioid	38	−10.6	−22.3	1.1	0.074
	Control	43				
Emotional well being	Opioid	38	−7.4	−17.8	3.0	0.162
	Control	43				
Social functioning	Opioid	38	−22.7	−36.4	−8.99	**0.002**
	Control	43				
Pain	Opioid	38	−19.5	−29.9	−8.98	**< 0.001**
	Control	43				
General health	Opioid	38	−7.0	−20.4	6.4	0.299
	Control	43				

*Note:* The estimate of the control group is set to be zero. Bold indicate significant differences (*p* < 0.05).

Abbreviations: CRT, continuous reaction time; DST, digit span test; HADS, Hospital Anxiety and Depression Scale; PSQI, Pittsburgh Sleep Quality Index; RAND SF‐36, 36‐Item Short Form Survey.

### 3.4 Outcomes Comparison Between Men in the Groups

Linear regression analysis comparing men in the opioid and control groups demonstrated significant differences in sleep, mood, and HRQoL. Men in L‐TOT had higher scores on the global sleep measure of PSQI (*p* = 0.01, moderate evidence) as well as on anxiety (*p* = 0.029, weak evidence) and depression (*p* = 0.040, weak evidence) scales of HADS. In addition, men in L‐TOT had lower scores in role physical (*p* = 0.038, weak evidence), role emotional (*p* < 0.001, strong evidence), fatigue (*p* = 0.020, weak evidence), emotional well‐being (*p* ≤ 0.001, strong evidence), social functioning (*p* ≤ 0.001, strong evidence) and pain (*p* = 0.014, weak evidence) of RAND SF‐36 compared to the control group (Table 4). These differences surpassed the MCID thresholds, demonstrating clinical significance.

**TABLE 4:** Adjusted linear regression analysis to compare outcomes between groups—only men.

Outcomes	Group	*n*	Estimate	95% CI		*p*
Sustained attention (CRT)						
10th percentile	Opioid	22	20.5	−28.3	69.4	0.399
	Control	18				
50th percentile	Opioid	22	40.6	−49.2	130.4	0.365
	Control	18				
90th percentile	Opioid	22	68.0	−88.9	225.0	0.385
	Control	18				
Short‐term/working memory (DST)						
Forward	Opioid	22	−0.6	−1.4	0.3	0.179
	Control	18				
Backward	Opioid	22	−0.5	−1.5	0.5	0.314
	Control	18				
Worst pain intensity	Opioid	22	1	−0.6	2.7	0.219
	Control	18				
Sleep (PSQI)						
Global score	Opioid	21	4.5	1.2	7.9	**0.010**
	Control	17				
Mood (HADS)						
Anxiety	Opioid	22	3.4	0.4	6.4	**0.028**
	Control	18				
Depression	Opioid	22	2.9	0.1	5.6	**0.040**
	Control	18				
Health‐related Quality of Life (RAND SF‐36)						
Physical functioning	Opioid	22	−12.1	−30.1	6.0	0.183
	Control	18				
Role physical	Opioid	22	−25.3	−49.2	−1.5	**0.038**
	Control	18				
Role emotional	Opioid	22	−47.2	−72.3	−22.2	**< 0.001**
	Control	18				
Fatigue	Opioid	22	−20.4	−37.3	−3.5	**0.019**
	Control	18				
Emotional well being	Opioid	22	−24.4	−37.8	−10.96	**0.001**
	Control	18				
Social functioning	Opioid	22	−34.4	−53.3	−15.5	**0.001**
	Control	18				
Pain	Opioid	22	−21.9	−38.9	−4.8	**0.014**
	Control	18				
General health	Opioid	22	−13.5	−33.4	6.4	0.177
	Control	18				

*Note:* The estimate of the control group is set to be zero. Missing data (control group): PSQI = 1. Missing data (opioid group): PSQI = 1. Bold indicate significant differences (*p* < 0.05).

Abbreviations: CRT, continuous reaction time; DST, digit span test; HADS, Hospital Anxiety and Depression Scale; PSQI, Pittsburgh Sleep Quality Index; RAND SF‐36, 36‐Item Short Form Survey.

### 3.5 Mediation Analyses With Endocrine Biomarkers

In the mediation analysis, PRL and IGF‐1 (SDS) were tested as potential mediators of the relationship between L‐TOT and sleep quality, physical functioning, social functioning, and pain (Table 5). Another mediation analysis was performed only for male patients, testing TT, fT, SHBG, DHEAS, and IGF‐1 as potential mediators of the relationship between L‐TOT and sleep quality, mood (anxiety and depression) role physical, role emotional, fatigue, emotional well‐being, social functioning, and pain (Table 6). According to the Bias‐Corrected Bootstrap analysis, only the association between L‐TOT and anxiety in men was significantly mediated by TT (natural indirect effect, *β* = 1.6, 95% CI: 0.1; 4.1, *p* = 0.045) (Table 6). A percentile Bootstrap analysis provided very similar 95% CIs, which did not affect the estimates and *p*‐values.

**TABLE 5:** Results of mediation analyses for all patients.

Outcomes	Group	*n*	Estimate	95% CI		*p*
PRL						
Sleep (PSQI)						
Global score	Opioid	35	2.6	0.2	4.9	0.034
	Control	41				
Health‐related Quality of Life (RAND SF‐36)						
Physical functioning	Opioid	36	−13.7	−25.8	−1.7	0.026
	Control	43				
Social functioning	Opioid	37	−20.2	−34.2	−6.3	0.005
	Control	43				
Pain	Opioid	37	−18.8	−29.7	−7.9	0.001
	Control	43				
IGF‐1 (SDS)						
Sleep (PSQI)						
Global score	Opioid	31	3.4	0.8	6.0	0.010
	Control	38				
Health‐related Quality of Life (RAND SF‐36)						
Physical functioning	Opioid	32	−16.1	−29.8	−2.5	0.021
	Control	40				
Social functioning	Opioid	33	−21.4	−36.9	−5.8	0.008
	Control	40				
Pain	Opioid	33	−20.2	−32.4	−8.0	0.002
	Control	39				

*Note:* The estimate of the control group is set to be zero.

Abbreviations: IGF‐1 (SDS), insulin‐like growth factor 1 standard deviation score; PRL, prolactin; PSQI, Pittsburgh Sleep Quality Index; RAND SF‐36, 36‐Item Short Form Survey.

**TABLE 6:** Results of mediation analyses including only men.

Dependent variable	Group	*n*	Estimate	95% CI		*p*	Natural indirect effect β (BCB 95% CI), *p*
TT							
Sleep (PSQI)							
Global score	Opioid	20	4.6	0.7	8.4	0.022	
	Control	17					
Mood (HADS)							
Anxiety	Opioid	21	1.7	−1.4	4.9	**0.271**	**1.6 (0.1; 4.1), *p* = 0.045**
	Control	18					
Depression	Opioid	21	2.9	−0.2	6.1	0.067	
	Control	18					
Health‐related Quality of Life (RAND SF‐36)							
Role physical	Opioid	21	−21.8	−48.96	5.43	**0.113**	−3.6 (−17.8; 9.7), *p* = 0.506
	Control	18					
Role emotional	Opioid	21	−39.0	−66.8	−11.2	0.008	
	Control	18					
Fatigue	Opioid	21	−20.3	−39.7	−1.0	0.040	
	Control	18					
Emotional well‐being	Opioid	21	−19.6	−34.4	−4.7	0.011	
	Control	18					
Social functioning	Opioid	21	−26.3	−46.8	−5.8	0.014	
	Control	18					
Pain	Opioid	21	−20.9	−40.5	−1.3	0.038	
	Control	18					
fT							
Sleep (PSQI)							
Global score	Opioid	20	4.5	0.1	8.9	0.045	
	Control	17					
Mood (HADS)							
Anxiety	Opioid	21	1.1	−2.6	4.8	**0.540**	2.3 (−0.42; 5.54), *p* = 0.042
	Control	18					
Depression	Opioid	21	3.9	0.3	7.4	0.033	
	Control	18					
Health‐related Quality of Life (RAND SF‐36)							
Role physical	Opioid	21	−27.5	−58.7	3.6	**0.081**	2.2 (−14.7; 18.1), *p* = 0.800
	Control	18					
Role emotional	Opioid	21	−40.6	−73.0	−8.3	0.016	
	Control	18					
Fatigue	Opioid	21	−26.2	−47.97	−4.5	0.020	
	Control	18					
Emotional well‐being	Opioid	21	−18.9	−36.1	−1.7	0.032	
	Control	18					
Social functioning	Opioid	21	−22.9	−46.5	0.7	**0.057**	−11.4 (−28.3; 4.1), *p* = 0.100
	Control	18					
Pain	Opioid	21	−25.1	−47.3	−2.8	0.029	
	Control	18					
SHBG							
Sleep (PSQI)							
Global score	Opioid	21	4.4	0.8	7.9	0.018	
	Control	18					
Mood (HADS)							
Anxiety	Opioid	21	4.0	0.8	7.2	0.015	
	Control	18					
Depression	Opioid	21	3.5	0.6	6.4	0.021	
	Control	18					
Health‐related Quality of Life (RAND SF‐36)							
Role physical	Opioid	21	−32.8	−57.4	−8.3	0.010	
	Control	18					
Role emotional	Opioid	21	−53.98	−79.97	−27.99	< 0.001	
	Control	18					
Fatigue	Opioid	21	−23.8	−41.7	−5.9	0.011	
	Control	18					
Emotional well being	Opioid	21	−27.0	−41.3	−12.8	0.001	
	Control	18					
Social functioning	Opioid	21	−37.4	−57.6	−17.3	0.001	
	Control	18					
Pain	Opioid	21	−25.1	−43.3	−6.8	0.01	
	Control	18					
DHEAS							
Sleep (PSQI)							
Global score	Opioid	20	4.3	0.5	8.0	0.027	
	Control	17					
Mood (HADS)							
Anxiety	Opioid	21	3.4	0.02	6.7	0.049	
	Control	18					
Depression	Opioid	21	3.5	0.5	6.5	0.022	
	Control	18					
Health‐related Quality of Life (RAND SF‐36)							
Role physical	Opioid	21	−25.6	−52.1	0.8	**0.058**	0.3 (−8.0; 16.0); *p* = 0.948
	Control	18					
Role emotional	Opioid	21	−49.9	−77.5	−22.4	0.001	
	Control	18					
Fatigue	Opioid	21	−22.2	−40.8	−3.6	0.021	
	Control	18					
Emotional well being	Opioid	21	−25.3	−40.2	−10.5	0.002	
	Control	18					
Social functioning	Opioid	21	−33.6	−54.5	−12.8	0.003	
	Control	18					
Pain	Opioid	21	−20.6	−39.6	−1.7	0.034	
	Control	18					
IGF‐1							
Sleep (PSQI)							
Global score	Opioid	20	4.5	0.8	8.2	0.020	
	Control	17					
Mood (HADS)							
Anxiety	Opioid	21	3.8	0.4	7.2	0.030	
	Control	18					
Depression	Opioid	21	4.3	1.5	7.2	0.004	
	Control	18					
Health‐related Quality of Life (RAND SF‐36)							
Role physical	Opioid	21	−30.8	−57.4	−4.2	0.025	
	Control	18					
Role emotional	Opioid	21	−57.1	−83.97	−30.22	< 0.001	
	Control	18					
Fatigue	Opioid	21	−23.8	−42.7	−5.0	0.015	
	Control	18					
Emotional well being	Opioid	21	−28.4	−43.1	−13.7	0.001	
	Control	18					
Social functioning	Opioid	21	−34.9	−56.2	−13.6	0.002	
	Control	18					
Pain	Opioid	21	−21.2	−40.5	−1.8	0.033	
	Control	18					

*Note:* The estimate of the control group is set to be zero. Bold numbers indicate significant different when controlling for mediator (*p* > 0.05). The bold number for the natural indirect effect indicate significant different if (*p* < 0.05).

Abbreviations: BCB 95% CI: bias‐corrected bootstrap 95% confidence interval; CRT: continuous reaction time; DHEAS: dehydroepiandrosterone; DST: digit span test; fT: free testosterone; HADS: Hospital Anxiety and Depression Scale; IGF‐1: insulin‐like growth factor 1; PSQI: Pittsburgh Sleep Quality Index; RAND SF‐36: 36‐Item Short Form Survey; SHBG: sex hormone binding globulin; TT: total testosterone.

## 4 Discussion and Conclusion

This study of 82 CNCP patients from a tertiary pain center demonstrated impairments in several parameters, which generally were worse in the group receiving opioids. The comparison between groups revealed that HRQoL domains (physical functioning, social functioning, and pain) were poorer in the opioid group. We have previously shown that opioid use could negatively affect the endocrine system, especially in male patients (Diasso et al. 2023). Men in the opioid group exhibited worse outcomes related to sleep quality, anxiety, depression, and six HRQoL domains (role physical, role emotional, fatigue, emotional well‐being, social functioning, and pain) compared to controls. Further, the association between L‐TOT and anxiety is possibly mediated by TT.

Studies regarding long‐term effects of L‐TOT on CNCP patient‐reported outcomes are sparse (Arteta et al. 2015; de Kleijn et al. 2022; Frers et al. 2021; Gros et al. 2013; Helmerhorst et al. 2014; Leung et al. 2022; Martins et al. 2012; Peng et al. 2015). This is a complex population with long pain trajectories, functional impairment, and polypharmacy (May et al. 2018; Zahlan et al. 2023). Therefore, we attempted to control for the most relevant confounders through strict inclusion and exclusion criteria in our study.

In a study of patients with fibromyalgia receiving opioids, poor improvement in pain‐related interference with insomnia, depression, daily activities, and functioning was found (Peng et al. 2015).

Complaints of sleep problems are frequent in CNCP patients and often aggravated by L‐TOT, although the evidence is inconsistent. Opioids can affect sleep in general, including sleep architecture and compromise breathing while sleeping (Cutrufello et al. 2020). L‐TOT may decrease slow‐wave sleep monitored by electroencephalography (EEG) and result in a moderate reduction in rapid eye movement (REM), monitored by electrooculogram (EOG) (Lintzeris et al. 2016; Shaw et al. 2005). An analysis of opioid‐naive patients compared with current and previous long‐term users revealed that the opioid effects on sleep can persist and be present during opioid withdrawal up to at least 6 months (Frers et al. 2021). In contrast to these findings, opioids have also been associated with improved sleep quality in CNCP patients, which could be explained by pain reduction (Busse et al. 2018).

Other investigations have described an association of L‐TOT with anxiety (Arteta et al. 2015; Gros et al. 2013; Helmerhorst et al. 2014; Leung et al. 2022; Martins et al. 2012) and depression (Grattan et al. 2012; Helmerhorst et al. 2014; Leung et al. 2022; Scherrer et al. 2017, 2020). Patients in opioid treatment who also exhibited psychological distress had more severe symptoms and disability compared with patients who did not take opioids. A randomized controlled trial reported a 50% incidence of mood disorders in patients prescribed opioids (Arkinstall et al. 1995). Additionally, other studies have shown significant associations between chronic pain and mood disorders (Jamison et al. 2011). These overlapping factors make it difficult to isolate the causal factors definitively. Opioids may affect brain function through the HPA‐axis and the hippocampus, leading to a reduced density of dendrites in the hippocampus, which may increase the risk of anxiety and depression (Lutz and Kieffer 2013; Robinson et al. 2002). Some researchers have reported adrenal insufficiency and reduced testosterone levels in opioid users, which may negatively affect mood and lead to the development of depression (Lutz and Kieffer 2013; Semenkovich et al. 2014). An American investigation also reported an increasing number of adults with opioid use disorders and co‐occurring anxiety and depressive disorders; in addition, approximately 54% of the sample were men (Ware et al. 2023). We have not explored opioid use disorder in this sample, but a systematic review indicated a prevalence of 34% in CNCP patients (Jantarada et al. 2021), which may also explain psychological distress in some of the patients.

It is well known that chronic pain affects the mesolimbic dopamine system negatively and thus results in a hypodopaminergic state, which can lead to reduced motivated behavior and thus an increased risk of developing anxiety and depression. Opioids usually stimulate dopamine secretion; however, L‐TOT is less effective in stimulating the mesolimbic dopamine neurons in chronic pain patients. This leads to a continued reduced reward response despite opioid treatment, which can lead to anhedonia and the development of depression (Taylor et al. 2016).

Pain interference with daily activities, emotional suffering, physical, social, and mental health, anxiety, and anger issues may lead to depression and contribute to lower HRQoL (Becker et al. 1997; Katz 2002; Paterniani et al. 2020; Radcliff et al. 2013; Sá et al. 2019; US Department of Health and Human Services 2019). Further, when CNCP patients start on opioids, additional interference with the endocrine system may worsen the patients' HRQoL (Abou‐Kassem et al. 2022; Højsted and Sjøgren 2007; Lennon et al. 2012; McGuirt and Brezing 2024). Animal studies have indicated that opioids may interfere with the endocrine system through central and peripheral pathways, involving the hypothalamus and the peripheral glands (Katz and Mazer 2009). However, studies in CNCP patients regarding opioid effects on endocrine function are few (Diasso et al. 2021) and mostly focused on the HPG axis. Opioids can decrease the pulsatile release of gonadotropin‐releasing hormone (GnRH) from the hypothalamus, consequently reducing the release of luteinizing hormone (LH) and follicle‐stimulating hormone (FSH) and thereby decreasing the synthesis of sex steroids by the gonads (McGuirt and Brezing 2024; Wehbeh and Dobs 2020). Furthermore, opioids can interfere with the secretion of PRL, inhibiting GnRH release and thus decreasing sex hormones (de Vries et al. 2020). Interestingly, one study showed that hypogonadism occurrence following L‐TOT was more frequent in men than in women (Fraser et al. 2009). Clinical symptoms like reduced libido and impotence were not assessed in our study, but studies have also found that they were more frequently reported by men than by women (Abs et al. 2000; Fraser et al. 2009; Reddy et al. 2010). Hypogonadal men experienced a negative effect on mood and reduced quality of life compared to controls (Horie 2010; Zitzmann 2020). However, our results regarding the potential mediation effects of endocrine parameters on the association between L‐TOT and health outcomes should be interpreted with caution. The cross‐sectional design and small sample size prevent establishing the required temporal sequence between exposure, mediator, and outcomes for mediation analysis. As a result, we cannot definitively determine whether endocrine biomarkers act as mediators. It is also possible that these endocrine parameters serve as effect modifiers or confounding factors. These significant findings should therefore be considered exploratory, and causal interpretations should be avoided. Most of the effects observed may be attributable to the direct impact of L‐TOT or a combination of direct and indirect effects. Future longitudinal studies are warranted to clarify the temporal relationships between variables, especially given the small sample size that may have been underpowered. Notably, the significant natural indirect effect of TT on anxiety in men was slightly significant, suggesting that some effects might arise from the direct opioids effect or a combined treatment effect including mediators.

This study is among the few assessing long‐term health effects of L‐TOT in CNCP patients and potential mediation by the endocrine system. Restrictive inclusion criteria minimized confounders, and bootstrapping refined mediation analysis. However, the cross‐sectional design limits causal interpretations, as associations observed likely relate to opioids' mode of action. The small sample size, calculated for the primary outcome, may be insufficient to detect true group differences in secondary outcomes. Additionally, participants were recruited from a single pain center, limiting the generalizability to broader populations, particularly in different healthcare settings or countries. Medication intake was not controlled, so it remains uncertain whether participants were actively taking opioids. Data relied on questionnaire responses, which are prone to recall bias. Due to the small sample size, analyses on the type, dose, and duration of opioid treatment were not feasible. L‐TOT was defined as stable daily opioid use for at least 4 weeks to facilitate the inclusion of CNCP patients in a clinical trial. Epidemiological studies, however, typically define L‐TOT as opioid use exceeding 3 months (Dowell et al. 2016; Hamina et al. 2022; Norwegian Ministry of Health and Care Services N 2007). The exclusion of patients on benzodiazepines is another limitation, as chronic pain patients often take other medications, including benzodiazepines. Consequently, our opioid group is a selected subset compared to the broader chronic pain population typically seen in clinical settings.

A clear association was observed between L‐TOT and worse scores on sleep quality, anxiety, depression, and HRQoL in CNCP patients. In the mediation analysis, a significant natural indirect effect of TT on anxiety was identified in men. However, the small sample size calls for caution regarding the interpretation. The direct effect of opioids or a total effect of the treatment may play a role. Future research should examine larger patient samples in longitudinal designs, include objective measures of patients' outcomes and opioid intake, and analyse effects by opioid type, dose, and duration of treatment to provide new and sound knowledge. Clinicians should be attentive to the development of opioid effects and regularly monitor CNCP patients in L‐TOT regarding these potential effects.

## Author Contributions

This study was designed by G.P.K., P.S., P.D.K.D., K.M.M., S.D.N. Acquisition of data was by P.D.K.D. and D.A.K. Planning of data analysis was by G.P.K., P.S., P.D.K.D., D.A.K., K.M.M. Interpretation of data and results was critically examined by all authors. D.A.K., G.P.K., P.S. had a primary role in preparing the manuscript draft. All authors revised the article draft and contributed to the final version. All authors have approved the final version of the manuscript and agreed to be accountable for all aspects of the work.

## Conflicts of Interest

The authors have no conflicts of interest. G.P.K. has received research grants from Novo Nordisk Foundation, European Commission, and the Danish Cancer Society to other projects.

## Supporting information


Data S1.

## Data Availability

Data are available upon reasonable request and are subjected to legal requirements.
