# Supplementary material for: Opioid Therapy in Chronic Pain: Assessment of Clinical Outcomes and Relationships With Endocrine Biomarkers
Source: Eur J Pain. 2025 Apr 29;29(6):e70027. doi: 10.1002/ejp.70027 (PMC12038877; doi:10.1002/ejp.70027)
Supplement: Supplementary file 1 — Data S1. [file EJP-29-0-s001.doc]

STROBE Statement—Checklist of items that should be included in reports of ***cross-sectional studies***

|  | Item No | Recommendation | Findings |
| --- | --- | --- | --- |
| **Title and abstract** | 1 | (*a*) Indicate the study’s design with a commonly used term in the title or the abstract | In the title page file (page 1) and the abstract file. |
|  |  | (*b*) Provide in the abstract an informative and balanced summary of what was done and what was found | In the abstract file. |
| Introduction | | |  |
| Background/rationale | 2 | Explain the scientific background and rationale for the investigation being reported | In the introduction section in the manuscript (page 1-2). |
| Objectives | 3 | State specific objectives, including any prespecified hypotheses | In the introduction section in the manuscript (page 1-2). |
| Methods | | |  |
| Study design | 4 | Present key elements of study design early in the paper | In the method section, section 2.1 in page 3 in the manuscript. |
| Setting | 5 | Describe the setting, locations, and relevant dates, including periods of recruitment, exposure, follow-up, and data collection | In the method section, section 2.1 in page 3 in the manuscript. |
| Participants | 6 | (*a*) Give the eligibility criteria, and the sources and methods of selection of participants | In the method section, section 2.2 in page 3 in the manuscript. |
| Variables | 7 | Clearly define all outcomes, exposures, predictors, potential confounders, and effect modifiers. Give diagnostic criteria, if applicable | In the method section, section 2.3 (page 4), section 2.3.1 (page 5), section 2.3.2 (page 5), section 2.3.3 (page 5), section 2.3.4 (page 6), section 2.3.5 (page 6), section 2.3.6 (page 6) in the manuscript. |
| Data sources/ measurement | 8* | For each variable of interest, give sources of data and details of methods of assessment (measurement). Describe comparability of assessment methods if there is more than one group | In the method section, section 2.3 (page 4), section 2.3.1 (page 5), section 2.3.2 (page 5), section 2.3.3 (page 5), section 2.3.4 (page 6), section 2.3.5 (page 6), section 2.3.6 (page 6) in the manuscript. |
| Bias | 9 | Describe any efforts to address potential sources of bias | In the method section, section 2.3.6 (page 6) and section 2.4 (page 6-7) in the manuscript. |
| Study size | 10 | Explain how the study size was arrived at | In the method section, section 2.2 (page 3), section 2.3 (page 4), section 2.3.6 (page 6), and section 2.4 (page 6-7) in the manuscript. |
| Quantitative variables | 11 | Explain how quantitative variables were handled in the analyses. If applicable, describe which groupings were chosen and why | In the method section, section 2.2 (page 3), section 2.3 (page 4), section 2.3.1 (page 5), section 2.3.2 (page 5), 2.3.3 (page 5), section 2.3.4 (page 6), 2.3.5 (page 6), section 2.3.6 (page 6), and section 2.4 (page 6-7) in the manuscript.  Figure 1 and figure 2 in the figures file. |
| Statistical methods | 12 | (*a*) Describe all statistical methods, including those used to control for confounding | In the method section, section 2.4 in page 6-7 in the manuscript.  Figure 1 and figure 2 in the figures file. |
|  |  | (*b*) Describe any methods used to examine subgroups and interactions | In the method section, section 2.4 in page 6-7 in the manuscript.  Figure 1 and figure 2 in the figures file. |
|  |  | (*c*) Explain how missing data were addressed | In the method section, section 2.4 in page 6-7 in the manuscript.  Figure 1 and figure 2 in the figures file. |
|  |  | (*d*) If applicable, describe analytical methods taking account of sampling strategy | In the method section, section 2.4 in page 6-7 in the manuscript.  Figure 1 and figure 2 in the figures file. |
|  |  | (*e*) Describe any sensitivity analyses | In the method section, section 2.4 in page 6-7 in the manuscript.  Figure 1 and figure 2 in the figures file. |
| Results | | |  |
| Participants | 13* | (a) Report numbers of individuals at each stage of study—eg numbers potentially eligible, examined for eligibility, confirmed eligible, included in the study, completing follow-up, and analysed | Table 1, table 2, table 3, table 4, table 5 and table 6 in the table file. |
|  |  | (b) Give reasons for non-participation at each stage | In the result section, section 3.3 (page 11), section 3.4 (page 12), and section 3.5 (page 13) in the manuscript.  Table 3, table 4, table 5, and table 6 in the table file. |
|  |  | (c) Consider use of a flow diagram |  |
| Descriptive data | 14* | (a) Give characteristics of study participants (eg demographic, clinical, social) and information on exposures and potential confounders | In the result section, section 3.1 (page 10), and section 3.2 (page 10) in the manuscript.  Table 1 and table 2 in the table file. |
|  |  | (b) Indicate number of participants with missing data for each variable of interest | In the method section, section 2.4 (page 6-7) in the manuscript. |
| Outcome data | 15* | Report numbers of outcome events or summary measures | In the result section, section 3.1 (page 10), section 3.2 (page 10), section 3.3 (page 11), section 3.4 (page 12), and section 3.5 (page 13) in the manuscript.  Table 1, table 2, table 3, table 4, table 5, and table 6 in the table file. |
| Main results | 16 | (*a*) Give unadjusted estimates and, if applicable, confounder-adjusted estimates and their precision (eg, 95% confidence interval). Make clear which confounders were adjusted for and why they were included | In the result section, section 3.3 (page 11), section 3.4 (page 12), and section 3.5 (page 13) in the manuscript.  Table 3, table 4, table 5, and table 6 in the table file. |
|  |  | (*b*) Report category boundaries when continuous variables were categorized | In the method section, section 2.4 (page 6-7) in the manuscript. |
|  |  | (*c*) If relevant, consider translating estimates of relative risk into absolute risk for a meaningful time period |  |
| Other analyses | 17 | Report other analyses done—eg analyses of subgroups and interactions, and sensitivity analyses | In the result section, section 3.5 (page 13) in the manuscript. |
| Discussion | | |  |
| Key results | 18 | Summarise key results with reference to study objectives | In the discussion section, page 16 in the manuscript. |
| Limitations | 19 | Discuss limitations of the study, taking into account sources of potential bias or imprecision. Discuss both direction and magnitude of any potential bias | In the discussion section, page 18 in the manuscript. |
| Interpretation | 20 | Give a cautious overall interpretation of results considering objectives, limitations, multiplicity of analyses, results from similar studies, and other relevant evidence | In the discussion section, page 16-19 in the manuscript. |
| Generalisability | 21 | Discuss the generalisability (external validity) of the study results | In the discussion section, page 18 in the manuscript. |
| Other information | | |  |
| Funding | 22 | Give the source of funding and the role of the funders for the present study and, if applicable, for the original study on which the present article is based | In the title page file. |

*Give information separately for exposed and unexposed groups.

**Note:** An Explanation and Elaboration article discusses each checklist item and gives methodological background and published examples of transparent reporting. The STROBE checklist is best used in conjunction with this article (freely available on the Web sites of PLoS Medicine at http://www.plosmedicine.org/, Annals of Internal Medicine at http://www.annals.org/, and Epidemiology at http://www.epidem.com/). Information on the STROBE Initiative is available at www.strobe-statement.org.
